# METTL3 promotes intrahepatic cholangiocarcinoma progression by regulating IFIT2 expression in an m^6^A-YTHDF2-dependent manner

**DOI:** 10.1038/s41388-022-02185-1

**Published:** 2022-01-29

**Authors:** Qiong-Cong Xu, Yi-Chih Tien, Yin-Hao Shi, Siyun Chen, Ying-Qin Zhu, Xi-Tai Huang, Chen-Song Huang, Wei Zhao, Xiao-Yu Yin

**Affiliations:** 1 Department of Pancreato-Biliary Surgery, The First Affiliated Hospital of Sun Yat-sen University, Guangzhou, 510080 China; 2 Key Laboratory of Stem Cells and Tissue Engineering (Sun Yat-sen University), Ministry of Education, Guangzhou, 510080 China; 3 Guangdong Provincial People’s Hospital, Guangdong Academy of Medical Sciences, Guangzhou, 510080 China

**Keywords:** Cancer genomics, Biliary tract cancer

## Abstract

N6-methyladenosine (m^6^A) RNA methylation has recently been found involving in regulatory mechanism of the tumor progression. Our aim was to explore the biological function and clinical significance of the m^6^A methyltransferase METTL3 in intrahepatic cholangiocarcinoma (ICC). In this study, we revealed that METTL3 was upregulated and predicted poor prognosis of patients with ICC. Multivariate regression analysis demonstrated that METTL3 expression was an independent predictor for overall survival in patients with ICC. Moreover, METTL3 knockdown inhibited ICC progression, while METTL3 overexpression showed the opposite effect. METTL3 inhibitor STM2457 also showed anti-tumor effect in ICC. Mechanistically, METTL3 transcription was driven by H3K4me3 activation. Upregulation of METTL3 mediated m^6^A modification of *IFIT2* mRNA and accelerated *IFIT2* mRNA decay in a YTHDF2-dependent manner, which promoted the development of ICC and lead to poorer prognosis. In summary, our findings revealed that H3K4me3 activation-driven *METTL*3 transcription promotes ICC progression by YTHDF2-mediated *IFIT2* mRNA degradation, suggesting that METTL3 may serve as a potential target for human ICC therapy.

## Introduction

Intrahepatic cholangiocarcinoma (ICC) is the second most common primary malignant liver cancer, which accounts for ~10% of all such cancers [1, 2]. The prognosis of patients with ICC is poor. Nearly 70% of patients with ICC are already unresectable at the time of diagnosis. Even in patients undergoing curative surgical treatment, the 5-year overall survival (OS) rate is ~30%, and the 5-year recurrence rate is up to 70% [3]. Therefore, effective systemic therapy during the course of the disease is required for the ICC. The combination of gemcitabine and cisplatin is the current first-line therapy for patients with unresectable ICC, but its efficacy remains very limited [4, 5]. Due to the lack of effective treatment and poor prognosis of ICC, understanding the molecular mechanisms underlying ICC development is urgently needed.

Accumulating evidence has revealed that ICC pathogenesis is complicated, which involves epigenetic, genetic, and proteomic alterations [6, 7]. Epigenetic regulation is one of the most common pathways causing gene aberrant expression and facilitating ICC progression [8]. N6-methyadenosine (m^6^A) modification is the most prevalent mRNA modification in eukaryote [9]. m^6^A modification is dynamic and reversible, which is regulated by m^6^A “writer” proteins (Methyltransferases like 3 [METTL3], Methyltransferases like 14 [METTL14] and Wilms tumor 1 associated protein [WTAP]) and “eraser” proteins (Fat-mass and obesity-associated protein [FTO] and Alkylation repair homolog protein 5 [ALKBH5]). In addition, specific “reader” proteins (YTH domain-containing proteins, YTHDF1-3, and YTHDC1-2) can recognize m^6^A sites and affect RNA process including mRNA stability, decay, splicing, and translation [10, 11]. Aberrant m^6^A modifications have been found to be involved in the carcinogenesis of a variety of human tumors [12]. However, the mechanism of carcinogenesis influenced by m^6^A modification dysregulation in ICC remains unclear.

Here, we dissected the reason for the dysregulation of METTL3 in ICC and revealed the regulatory mechanism of the m^6^A modification mediated by METTL3 in ICC. We also demonstrated that METTL3 regulates IFIT2 expression in an m^6^A-YTHDF2-dependent manner, and METTL3 may be a novel prognostic predictor and therapeutic target for ICC.

## Results

### METTL3 expression was elevated in ICC and associated with poorer prognosis

**Fig. 1:**
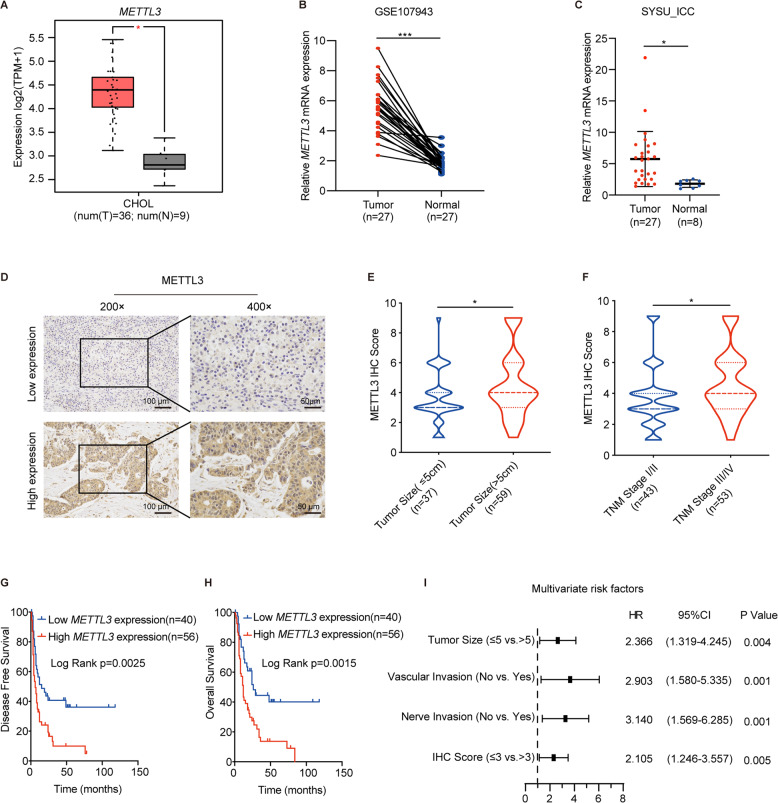
Elevated METTL3 expression is correlated with poorer prognosis of ICC patients. **A** *METTL3* expression of ICC tumor (*n* = 36) and normal tissue (*n* = 9) in GEPIA2 database. B *METTL3* expression of ICC tumor (*n* = 27) and normal tissue (*n* = 27) in public dataset GSE107943. **C** *METTL3* expression in ICC tumor (*n* = 27) and adjacent normal bile duct tissue (*n* = 8) were measured by real-time quantitative reverse transcription polymerase chain reaction (qRT-PCR). **D** Representative immunohistochemistry (IHC) staining images of ICC tumors expressing low or high levels of METTL3. **E** Correlation analysis of METTL3 expression with tumor size (≤5 cm vs. >5 cm). **F** Correlation analysis of METTL3 expression with tumor, node, metastasis stages (stage I/II vs. III/IV). **G** Kaplan–Meier survival curves of disease-free survival (DFS) in 96 ICC patients, stratified by METTL3 IHC score (METTL3 low expression, *n* = 40 vs. METTL3 high expression, *n* = 56). The *P* value was calculated using the log-rank test. **H** Kaplan–Meier survival curves of overall survival (OS) in 96 ICC patients, stratified by METTL3 IHC score (METTL3 low expression, *n* = 40 vs. METTL3 high expression, *n* = 56). The *P* value was calculated using the log-rank test. **I** Multivariable analyses for overall survival were performed in the ICC cohort. *P < 0.05, ***P < 0.001, according to Student’s *t* test.

The m^6^A levels are mainly regulated by m^6^A writers and erasers. Therefore, we detected mRNA expression of m^6^A modulators in ICC tissues. The results showed that methyltransferase METTL3 was significantly up-regulated in all ICC data sets, including GEPIA2 dataset (Fig. 1A), GSE107943 (Fig. 1B), and our dataset (Fig. 1C). We then measured the expression level of METTL3 in tumor tissues of 96 ICC patients by immunohistochemistry (IHC) staining (Fig. 1D) and compared the correlation between METTL3 expression and clinical characteristics (Supplementary Table 1). METTL3 expression was positively correlated with tumor size (*P* < 0.05) (Fig. 1E) and tumor, node, metastasis (TNM) stage (*P* < 0.05) (Fig. 1F). Kaplan–Meier analysis showed that ICC patients with high METTL3 expression had poorer DFS (*P* = 0.0025, Fig. 1G) and OS (*P* = 0.0015, Fig. 1H). Univariate regression analysis illustrated that tumor size, vascular invasion, nerve invasion, TNM stage, lymphatic metastasis, distant metastasis, and METTL3 expression were associated with OS in 96 ICC patients, and Multivariate regression analysis demonstrated that METTL3 expression was an independent predictor for overall survival in patients with ICC (HR = 2.105, 95% CI [1.246–3.557] (Supplementary Table 2 and Fig. 1I)). These results suggest that METTL3 is upregulated in ICC and might be an independent prognostic marker for ICC patients.

### METTL3 transcription is activated by H3K4me3 in ICC

**Fig. 2:**
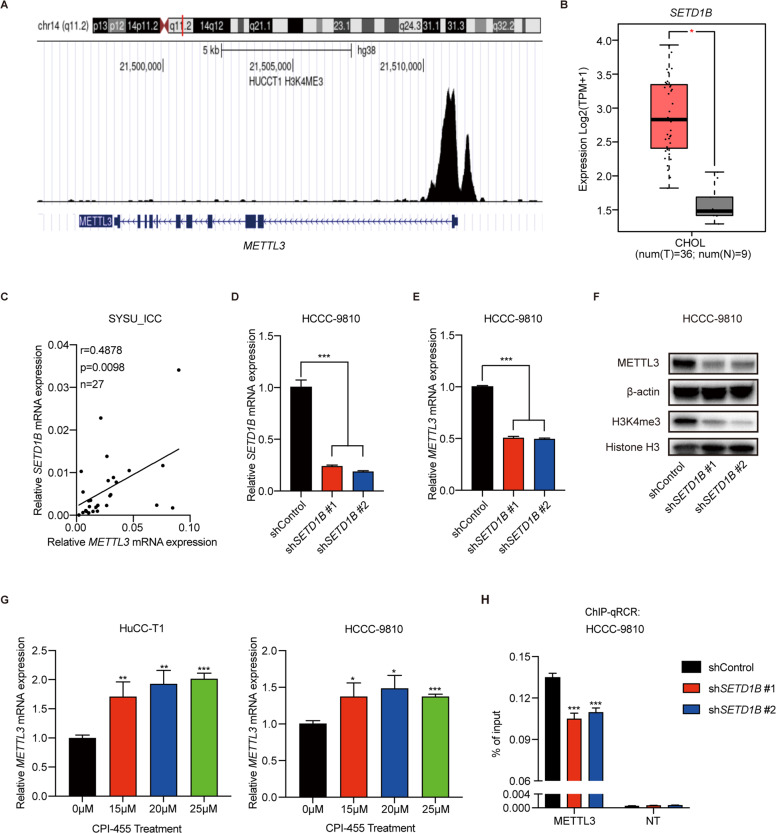
H3K4me3 activates METTL3 transcription in ICC. **A** Analysis of H3K4me3 modification in the *METTL3* locus in HuCC-T1 cell (datasets from the Cistrome Data Browser). **B** *SETD1B* expression in ICC tumors (*n* = 36) and normal tissues (*n* = 9) in the GEPIA2 database. **C** Correlation analysis of *METTL3* expression with *SETD1B* expression in our ICC cohort. **D** The mRNA levels of *SETD1B* in *SETD1B*-KD and control HCCC-9810 cells were confirmed by RT-qPCR. **E** The mRNA levels of *METTL3* in *SETD1B*-KD and control HCCC-9810 cells were confirmed by RT-qPCR. **F** The protein levels of METTL3 and H3K4me3 modification after *SETD1B* silencing in HCCC-9810 cells were confirmed by western blotting. **G** The mRNA level of *METTL3* after CPI-455 treatment for 72 h in HuCC-T1 and HCCC-9810 cells was confirmed by RT-qPCR. **H** Chromatin immunoprecipitation-qPCR analysis of H3K4me3 enrichment in the *METTL3* locus or a flank region with no signal in *SETD1B*-KD or control HCCC-9810 cells. NT = A flank region with no signal was used as a negative control. The results are presented as mean ± SD of three independent experiments. *P < 0.05, **P < 0.01, ***P < 0.001, according to Student’s *t* test.

Epigenetic transcriptional activation is an important regulatory pathway of gene transcription. We first analyzed chromatin modification at the transcription start site of *METTL3* using the Cistrome Data Browser (http://cistrome.org/). As shown in Fig. 2A, tri-methylation of lysine 4 on histone 3 (H3K4me3) is enriched in the transcription start site of *METTL3*, indicating that H3K4me3 might regulate the expression of METTL3. SETD1B is a common transcriptional activator with H3K4 tri-methyltransferase activity. Analysis of GEPIA2 data revealed that *SETD1B* mRNA expression was significantly upregulated in ICC (Fig. 2B) and correlated with *METTL3* mRNA expression (Supplementary Fig. 1). The results from our ICC cohort also showed that *SETD1B* mRNA expression was positively correlated with *METTL3* mRNA expression (Fig. 2C). We then knocked down *SETD1B* mRNA using specific shRNA (Fig. 2D) and found that knockdown of *SETD1B* mRNA significantly reduced *METTL3* mRNA levels (Fig. 2E). The protein levels of METTL3 and H3K4me3 were also reduced (Fig. 2F). Next, we treated ICC cells with CPI-455, a specific KDM5 inhibitor, which can improve the whole level of H3K4me3 modification. The results showed that *METTL3* expression significantly increased in a dose-dependent manner (Fig. 2G). Moreover, the chromatin immunoprecipitation assay showed that H3K4me3 signals were enriched in the transcription start site of *METTL3*, and knockdown of *SETD1B* could significantly decrease the enrichment of H3K4me3 (Fig. 2H). These results showed that the increase of METTL3 expression may partly be attributed to H3K4me3 enrichment at the transcription start site of *METTL3*.

### Targeting of METTL3 suppresses ICC progression

**Fig. 3:**
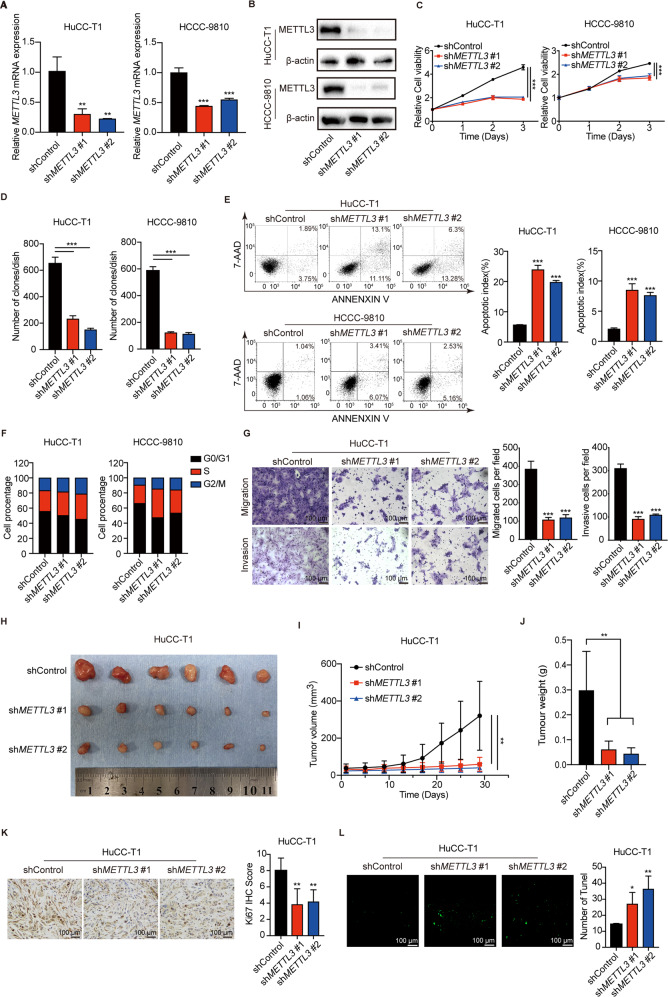
METTL3 knockdown inhibits ICC progression. **A** The mRNA level of *METTL3* after *METTL3* silencing in HuCC-T1 and HCCC-9810 cells was confirmed by RT-qPCR. **B** The protein level of METTL3 after *METTL3* silencing in HuCC-T1 and HCCC-9810 cells was confirmed by Western blotting. **C** Cell growth curve of HuCC-T1 and HCCC-9810 cells transfected with *METTL3* shRNA or Control. **D** Colony-forming assays after *METTL3* silencing in HuCC-T1 and HCCC-9810 cells. **E** Apoptosis analysis of HuCC-T1 and HCCC-9810 cells transfected with shControl or sh*METTL3*. **F** Cell cycle analysis of HuCC-T1 and HCCC-9810 cells transfected with shControl or sh*METTL3*. **G** Cell migration ability and cell invasion ability after sh*METTL3*-transfection in HuCC-T1 cell. **H** Xenograft tumors in each group were shown. The mice were sacrificed 28 days post-injection. **I** Tumor growth curves after the injection of sh*METTL3* and Control HuCC-T1 cells. Tumor volume was calculated every 4 days. **J** Tumor weight of sh*METTL3* and Control groups was measured. **K** Representative IHC staining of Ki67 in tumors with different treatments. **L** Representative images of TUNEL analysis in tumors with different treatments. The results are presented as mean ± SD of three independent experiments. *P < 0.05, **P < 0.01, ***P < 0.001, according to a Student’s *t* test.

To determine the biological role of METTL3 in ICC progression, stable *METTL3*-knockdown ICC cells were established. The knockdown efficiency of *METTL3* was verified by qPCR (Fig. 3A) and western blotting (Fig. 3B). Knockdown of *METTL3* significantly inhibited ICC cells proliferation (Fig. 3C) and colony formation ability (Fig. 3D and Supplementary Fig. 2A). METTL3 knockdown also significantly promotes ICC cells apoptosis (Fig. 3E) and arrested the cell cycle in S phase (Fig. 3F and Supplementary Fig. 2C). Migration and invasion assays showed that silencing *METTL3* significantly inhibited the ability of migration and invasion in ICC cells (Fig. 3G and Supplementary Fig. 2B). Next, *METTL3*-knockdown HuCC-T1 and HCCC-9810 cells and control cells were subcutaneously injected into mice. The *METTL3*-knockdown tumors grew more slowly than the tumors in the control group (Fig. 3H and Supplementary Fig. 2D). The tumor volume was calculated every 4 days (Fig. 3I and Supplementary Fig. 2E). The tumor weight was markedly lower in the *METTL3*-knockdown group compared to the control group (Fig. 3J and Supplementary Fig. 2F). In addition, IHC results showed Ki-67 in *METTL3*-knockdown tumors was significantly decreased compared with that in the control group (Fig. 3K and Supplementary Fig. 2G). TUNEL assay revealed that more apoptotic cells were found in METTL3-knockdown tumors (Fig. 3L and Supplementary Fig. 2H).

Recently, a novel and selective METTL3 inhibitor, STM2457 has shown therapeutic effect in acute myeloid leukemia [13]. Here, we examine the effect of STM2457 on ICC progression. As expected, STM2457 can inhibit the proliferation (Supplementary Fig. 3A) of ICC cells in a dose-dependent manner. In addition, STM2457 treatment can promote apoptosis of ICC cells (Supplementary Fig. 3B) and arrest the cell cycle of ICC cells in S phase (Supplementary Fig. 3C). Furthermore, STM2457 treatment also inhibited cell invasion and migration of ICC cells in a dose-dependent manner (Supplementary Fig. 3D). These results suggest that therapeutically targeting METTL3 may be a promising treatment for ICC.

### METTL3 overexpression promotes ICC progression

**Fig. 4:**
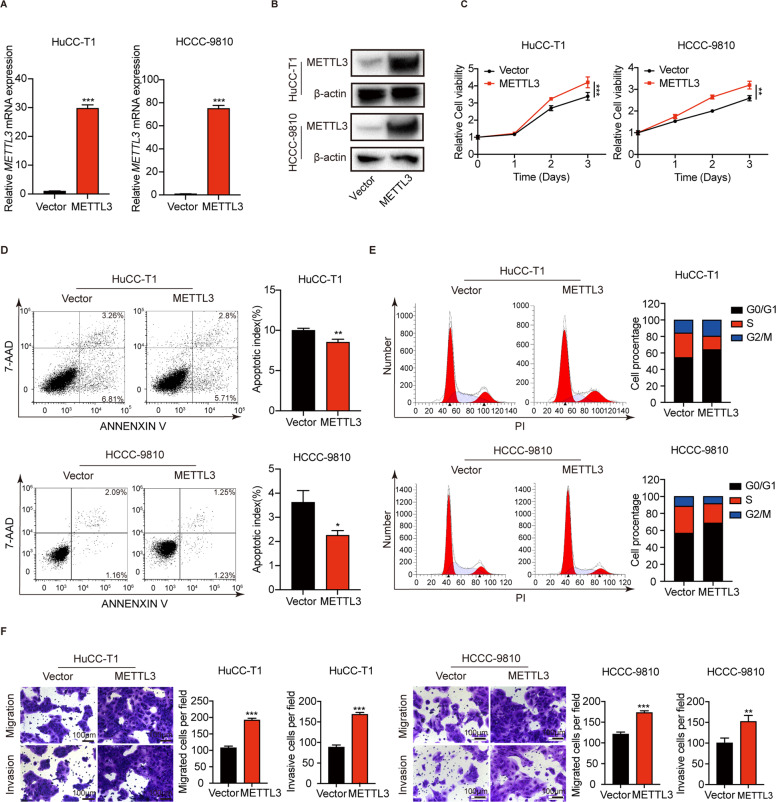
METTL3 overexpression promotes ICC progression. **A** The mRNA level of *METTL3* after METTL3 overexpression in HuCC-T1 and HCCC-9810 cells was confirmed by RT-qPCR. **B** The protein level of METTL3 after METTL3 overexpression in HuCC-T1 and HCCC-9810 cells was confirmed by Western blotting. **C** Cell growth curve of HuCC-T1 and HCCC-9810 cells transfected with METTL3-OE or Vector. **D** Apoptosis analysis of HuCC-T1 and HCCC-9810 cells transfected with METTL3-OE or Vector. **E** Cell cycle analysis of HuCC-T1 and HCCC-9810 cells transfected with METTL3-OE or Vector. **F** Cell migration ability and cell invasion ability of HuCC-T1 and HCCC-9810 cells transfected with METTL3-OE or Vector. The results are presented as mean ± SD of three independent experiments. *P < 0.05, **P < 0.01, ***P < 0.001, according to a Student’s *t* test.

To test the effect of METTL3 overexpression on ICC progression, we also established stable *METTL3*-overexpressing ICC cells. The efficiency of overexpression was verified by qPCR (Fig. 4A) and western blotting (Fig. 4B). Overexpression of METTL3 significantly promotes ICC cells proliferation (Fig. 4C). METTL3 overexpression have mild effect in inhibiting ICC cells apoptosis (Fig. 4D). In addition, METTL3 overexpression reduced the arrest of the cell cycle in S phase (Fig. 4E). Migration and invasion assays showed that METTL3 overexpression significantly promotes the ability of migration and invasion in ICC cells (Fig. 4F). These results suggest that METTL3 is essential in promoting ICC progression.

### METTL3 facilitates the ICC progression by downregulating IFIT2 expression

**Fig. 5:**
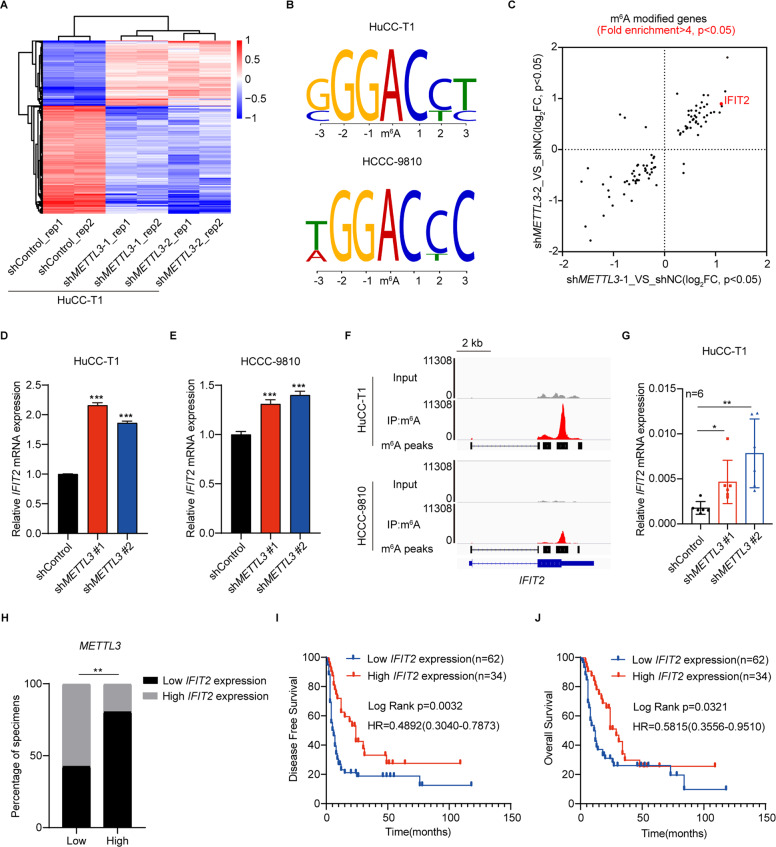
IFIT2 is the downstream target of METTL3. **A** Heatmap showing the expression changes of *METTL3*-KD and Control HuCC-T1 cell. **B** The m^6^A consensus sequence motifs in HuCC-T1 and HCCC-9810 cells was identified. **C** Scatter plot shows the genes with m^6^A modification (fold enrichment >4 and *P* < 0.05) in both HuCC-T1 and HCCC-9810 cells and differentially expressed (Log_2_foldchange > 0.5 and *P* < 0.05) in HuCC-T1 cells after METTL3 silencing. **D** Relative expression of *IFIT2* in *METTL3*-KD and Control HuCC-T1 cells. **E** RT-qPCR analysis of *IFIT2* after *METTL3* silencing in HCCC-9810 cells. **F** Integrative genomics viewer (IGV) plots indicates m^6^A peaks at *IFIT2* mRNAs in MeRIP-seq of ICC cells. The *y* axis shows the sequence read number, and the blue boxes represent protein-coding exons. **G** RT-qPCR analysis of *IFIT2* mRNA expression in HuCC-T1 xenograft models after METTL3 knockdown or not. **H** Bar graphs indicate the correlation of METTL3 expression with IFIT2 expression in ICC specimens. **I** Kaplan–Meier survival curves of DFS in 96 patients with ICC. (IFIT2 low expression, *n* = 62 vs. IFIT2 high expression, *n* = 34). The *P* value was calculated using the log-rank test. HR hazard ratio. **J** Kaplan–Meier survival curves of OS in 96 patients with ICC. (IFIT2 low expression, *n* = 62 vs. IFIT2 high expression, *n* = 34). The *P* value was calculated using the log-rank test. HR hazard ratio. The results are presented as mean ± SD of three independent experiments. *P < 0.05, **P < 0.01, ***P < 0.001, according to Student’s *t* test.

To delineate the molecular mechanism by which METTL3 accelerates ICC progression, RNA-seq in HuCC-T1 cell with or without *METTL3* knockdown was performed (Fig. 5A). RNA-seq revealed that 294 transcripts were significantly upregulated upon *METTL3* knockdown (Supplementary Fig. 4A), while 488 transcripts were significantly downregulated upon *METTL3* knockdown (Supplementary Fig. 4B). We then performed Methylated RNA immunoprecipitation-seq (MeRIP-seq) in HuCC-T1 and HCCC-9810 cells. The motifs of m^6^A peaks were consistent with the consensus sequence of RRACH (R = G/A, H = A/C/U) (Fig. 5B). There were 28 genes with m^6^A modification (fold enrichment >4 and *P* < 0.05) in both HuCC-T1 and HCCC-9810 cells and were differentially expressed (Log_2_foldchange > 0.5 and *P* < 0.05) in *METTL3* knockdown HuCC-T1 cell (Fig. 5C and Supplementary Fig. 4C). IFIT2 has been reported as a tumor suppressor for several tumor types [14, 15]. Among all the genes, IFIT2 is one of the top up-regulated genes in METTL3 knockdown cells comparing with shControl. The RPKM value of *IFIT2* in RNA-seq is shown in Fig. 5D. The mRNA levels of *IFIT2* also markedly increased following *METTL3* knockdown in another ICC cell line, HCCC-9810 (Fig. 5E). Moreover, the m^6^A peak in IFIT2 3’UTR is very prominent in MeRIP-seq of both HuCC-T1 and HCCC-9810 cells (Fig. 5F). Then, we measured the mRNA expression of IFIT2 in xenograft tumors. The results showed that the expression of IFIT2 was higher in METTL3 knockdown HuCC-T1 and HCCC-9810 xenograft tumors compared to the control ones (Fig. 5G and Supplementary Fig. 4D). We further measured the protein level of IFIT2 in the tumor tissues of 96 ICC patients by IHC staining (Supplementary Fig. 4E). The expression of IFIT2 in 96 ICC patients was negatively correlated with the expression of METTL3 (Fig. 5H). Kaplan–Meier analysis revealed that ICC patients with low IFIT2 expression had poorer DFS (*P* = 0.0032, Fig. 5I) and OS (*P* = 0.0321, Fig. 5J).

**Fig. 6:**
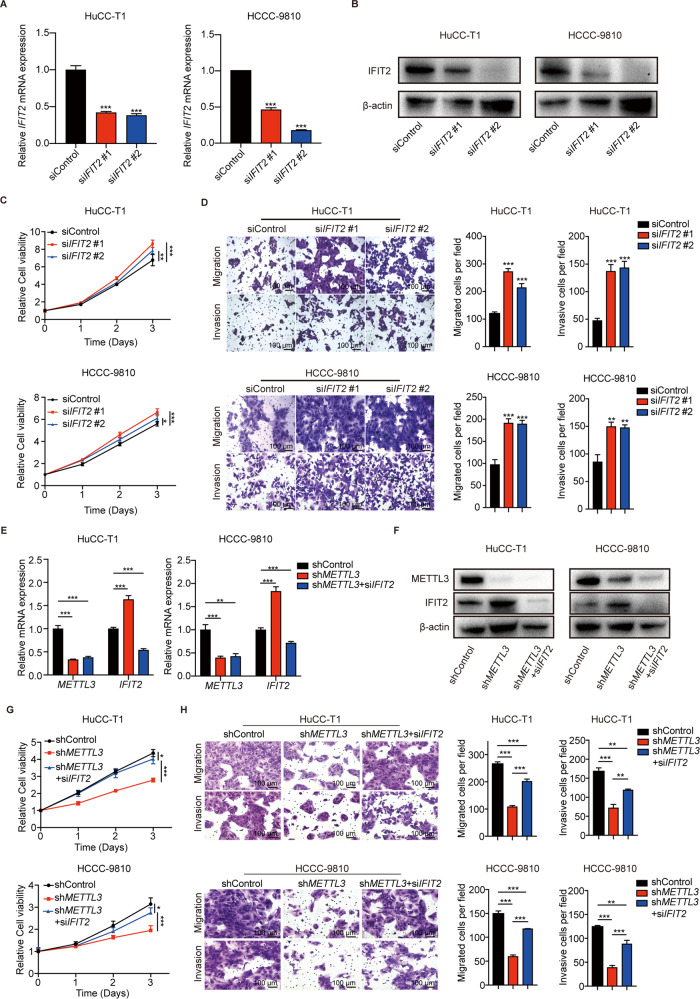
METTL3 accelerates the ICC progression by downregulating IFIT2 expression. **A** The mRNA level of *IFIT2* after *IFIT2* silencing in HuCC-T1 and HCCC-9810 cells was confirmed by RT-qPCR. **B** The protein level of IFIT2 after *IFIT2* silencing in HuCC-T1 and HCCC-9810 cells was confirmed by western blotting. **C** Cell growth curve of HuCC-T1 and HCCC-9810 cells transfected with *IFIT2* siRNA or Control. **D** Cell migration ability and cell invasion ability after *IFIT2* silencing in HuCC-T1 and HCCC-9810 cells. **E** The mRNA level of *METTL3* and *IFIT2* after *METTL3* or /and *IFIT2* silencing in HuCC-T1 and HCCC-9810 cells was confirmed by RT-qPCR. **F** The protein level of METTL3 and IFIT2 after *METTL3* or /and *IFIT2* silencing in HuCC-T1 and HCCC-9810 cells was confirmed by western blotting. **G** Cell growth curve of HuCC-T1 and HCCC-9810 cells after *METTL3* or /and *IFIT2* silencing. **H** Cell migration ability and cell invasion ability in HuCC-T1 and HCCC-9810 cells after *METTL3* or /and *IFIT2* silencing. The results are presented as mean ± SD of three independent experiments. *P < 0.05, **P < 0.01, ***P < 0.001, according to Student’s *t* test.

We then designed two siRNAs targeting *IFIT2* and verified the knockdown efficiency by RT-qPCR and western blotting (Figs. 6A, B). Knockdown of IFIT2 have mild effect in promoting cell proliferation of ICC cells (Fig. 6C), and dramatically promoted cell migration, and invasion (Fig. 6D). Then, the expression of *IFIT2* was knocked down in *METTL3* stable knockdown ICC cells using specific siRNAs (Figs. 6E, F). As expected, knockdown of *IFIT2* rescued the proliferation (Fig. 6G), migration, and invasion ability (Fig. 6H) of *METTL3* knockdown ICC cells. Our data suggest that METTL3 promotes ICC progression by downregulating the expression of IFIT2.

### METTL3 reduced *IFIT2* mRNA stability through an m^6^A-YTHDF2-dependent pathway

**Fig. 7:**
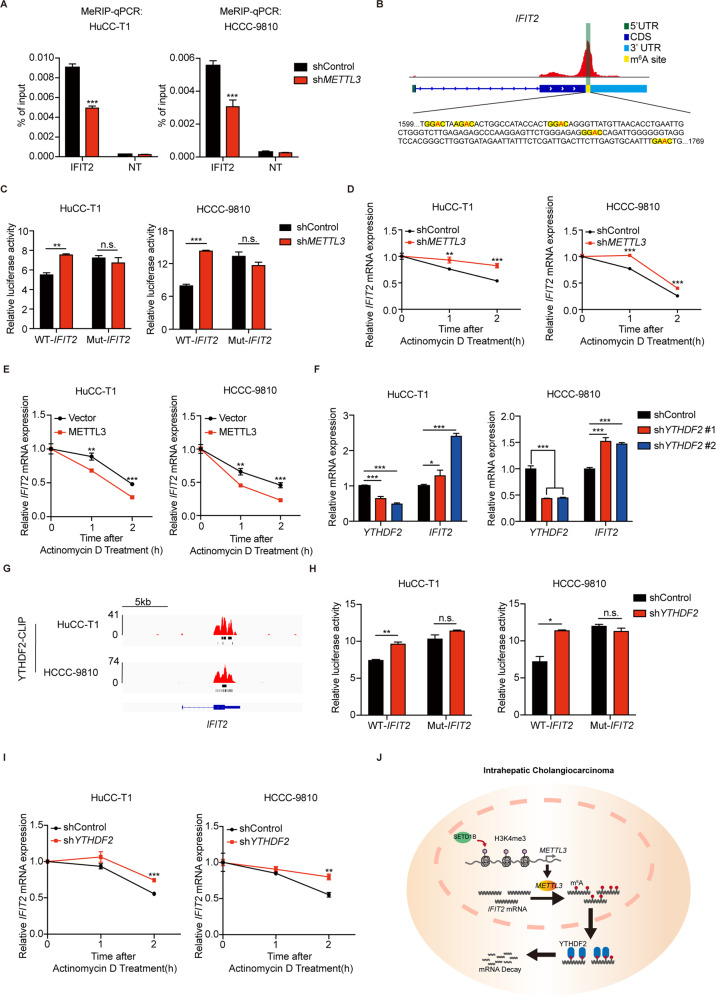
METTL3 reduced IFIT2 mRNA stability through an m^6^A-YTHDF2-dependent pathway. **A** MeRIP-qPCR analysis of m^6^A enrichment in the IFIT2 locus in METTL3-KD or Control HuCC-T1 and HCCC-9810 cells. NT = A flank region with no signal was used as a negative control. **B** Schematic representation of mutated (RRAC to RRCC) 3′UTR of psiCHECK2 vector to investigate the roles of m^6^A in 3′UTR in IFIT2 expression. **C** Relative luciferase activity of *IFIT2* 3’UTR with wild-type or mutated m^6^A sites after *METTL3* silencing in HuCC-T1 and HCCC-9810 cells. Renilla luciferase activity was measured and normalized to firefly luciferase activity. **D** RT-qPCR analysis of *IFIT2* after actinomycin D treatment in sh*METTL3* or Control HuCC-T1 and HCCC-9810 cells. **E** RT-qPCR analysis of *IFIT2* after actinomycin D treatment in *METTL3*-OE or Vector HuCC-T1 and HCCC-9810 cells. **F** The mRNA level of *YTHDF2* and *IFIT2* after *YTHDF2* silencing in HuCC-T1 and HCCC-9810 cells was confirmed by RT-qPCR. **G** IGV plots indicates YTHDF2 binding at *IFIT2* mRNAs. The *y* axis shows the sequence read number, and the blue boxes represent protein-coding exons. **H** Relative luciferase activity of *IFIT2* 3’UTR with wild-type or mutated m^6^A sites after YTHDF2 silencing in HuCC-T1 and HCCC-9810 cells. Renilla luciferase activity was measured and normalized to firefly luciferase activity. **I** RT-qPCR analysis of *IFIT2* after actinomycin D treatment in sh*YTHDF2* or Control HuCC-T1 and HCCC-9810 cells. **J** The schematic diagram for function and mechanism of METTL3 in ICC. The results are presented as mean ± SD of three independent experiments. *P < 0.05, **P < 0.01, ***P < 0.001, according to Student’s *t* test.

METTL3 usually acts as an oncogene by catalyzing the m^6^A modification of target genes [16]. To confirm that METTL3 catalyzes the m^6^A modification of *IFIT2*, MeRIP-qPCR was performed. The results showed that the m^6^A modification significantly enriched in *IFIT2* mRNA, but decreased in *METTL3* knockdown HuCC-T1 and HCCC-9810 cells (Fig. 7A). We then constructed dual luciferase reporter plasmids containing wild-type and mutant 3’ UTR of *IFIT2* and relative luciferase activity was compared in both HuCC-T1 and HCCC-9810 cells. The specific mutation position information of the reporter plasmid used is shown in Fig. 7B. Knockdown *METTL3* could affect the luciferase activity of the wild-type *IFIT2*-fused reporter but not the reporter with mutation on the m^6^A consensus sequences (Fig. 7C). HuCC-T1 and HCCC-9810 cells were treated with a transcription inhibitor (Actinomycin D) for the indicated times. Decelerated mRNA decay was observed upon knockdown of *METTL3* (Fig. 7D), while overexpression of *METTL3* showed the opposite effect (Fig. 7E). As previously reported [10], YTHDF2 could induce target mRNA degradation by reading m^6^A modification sites. Therefore, we tested whether YTHDF2 is involved in the regulation of *IFIT2* mRNA stability. The mRNA levels of *IFIT2* also markedly increased following *YTHDF2* knockdown in both HuCC-T1 and HCCC-9810 cells (Fig. 7F). CLIP-seq showed that YTHDF2 can bind to the m^6^A modification site of *IFIT2* in both HuCC-T1 and HCCC-9810 cells (Fig. 7G). Further dual luciferase activity assay also showed that the luciferase activity of wild-type *IFIT2*-fused reporter was obviously augmented upon YTHDF2 knockdown, but exhibiting no difference in mutation group (Fig. 7H). The actinomycin D assay also showed that knockdown of YTHDF2 could decelerate the mRNA decay of *IFIT2* (Fig. 7I), and METTL3 knockdown has no significant effect on decelerating the mRNA decay of IFIT2 in ICC cells with YTHDF2 knockdown (Supplementary Fig. 5). Together, our results showed that METTL3-mediated reduced *IFIT2* mRNA stability in a YTHDF2-dependent manner.

## Discussion

The m^6^A modification is the most prevalent mRNA modification in eukaryote. m^6^A modification has been reported involving in a variety of human diseases, including cardiovascular disease, metabolic disease, viral infection, and cancer progression [17]. m^6^A modification has shown potential in cancer by regulating mRNA decay, translation, and processing. The dysregulation of “writer,” “eraser,” and “reader” protein of m^6^A modification has been found in various human cancers and promotes cancer progression. However, the role and effect of m^6^A modification in ICC remain unclear. In this study, we first found that the major m^6^A writer METTL3 was upregulated in ICC. Mechanistically, H3K4me3 activation-driven *METTL3* transcription mediated m^6^A modification of *IFIT2* mRNA and accelerated *IFIT2* mRNA decay in a YTHDF2-dependent manner. The decreased mRNA levels of *IFIT2* promote the progression of ICC and lead to worse clinical prognosis (Fig. 7J).

METTL3 is the major component of the m^6^A methyltransferase complex. The aberrant transcription of METTL3 has been reported in various cancer types [16]. Previous studies have shown that METTL3 attributes to the oncogenesis of bladder cancer [18], acute myeloid leukemia [19], lung cancer [20], liver cancer [21], breast cancer [22], and gastric cancer [23]. However, the mechanisms for the dysregulation of METTL3 is still unclear. Wang et al. found that *METTL3* transcription was activated by H3K27ac modification in gastric cancer [23]. In the present study, we first found that knockdown of tri-methyltransferase decreased the enrichment of H3K4me3 in *METTL3* and inhibited the mRNA expression of *METTL3*. In contrast, KDM5 inhibitors can increase the enrichment of H3K4me3 in *METTL3* and increase *METTL3* transcription, suggesting that H3K4me3 modification mediated *METTL3* transcription in ICC. Loss-of-function and gain-of-function experiments demonstrated the essential role of METTL3 in promoting ICC progression. The METTL3 inhibitor STM2457 also exhibits anti-tumor effect in ICC. Thus, therapeutically targeting METTL3 may be a promising treatment for ICC.

One of the most important functions of m^6^A modification is epigenetically silencing tumor suppressor genes. Interferon (IFN)-induced protein with tetratricopeptide repeats 2 (IFIT2), also known as ISG54, is a member of the IFN-stimulated genes (ISGs). IFIT2 is a direct response target to type I IFNs (IFN-Is) and plays an important role in the innate immune response [24]. IFIT2 has been identified as a tumor suppressor in several tumor types, such as oral squamous cell carcinoma, breast cancer, gallbladder cancer, and lung cancer [25–28]. RNA-seq and rescue assays illustrated that *IFIT2* was one of the key regulatory targets of METTL3 in ICC. m^6^A modification relies on reader proteins that binds to m^6^A modified sites to exert their biological functions. mRNA decay of tumor suppressor genes in cancer progression is mainly mediated by YTHDF2. Wong et al. reported that METTL3 promoted hepatocellular carcinoma carcinogenesis through YTHDF2-dependent post-transcriptional silencing of SOCS2 [21]. Xie et al. reported that YTHDF2 promotes prostate cancer progression by mediating the mRNA degradation of *LHPP* and *NKX3-1* in an m^6^A-dependent manner and by inducing AKT phosphorylation [29]. In this study, we revealed that METTL3 silenced *IFIT2* expression through an m^6^A-YTHDF2-dependent mechanism. In addition, IFIT2 was shown to be a novel prognostic predictor for ICC.

In conclusion, our study reveals that elevated METTL3 expression correlated with poor prognosis in patients with ICC and plays an oncogenic role in ICC progression. Mechanistically, we found that H3K4me3 activation-driven transcription is the reason for the dysregulation of METTL3 in ICC. METTL3 regulates IFIT2 expression in an m^6^A-YTHDF2-dependent manner. Therefore, METTL3 may be a novel predictor and therapeutic target for ICC.

## Materials and methods

### Patients’ specimens

The paraffin-embedded specimens of 96 ICC patients who underwent surgical resection were obtained from the pathology department of the First Affiliated Hospital of Sun Yat-sen University (Guangzhou, China) and used for subsequent immunohistochemical experiments of METTL3 and IFIT2 protein expression. The clinical information of 96 ICC patients were shown in Supplementary Table 3. Another 27 ICC tumor tissues and 8 adjacent normal bile duct tissues for subsequent quantitative real-time PCR of *METTL3* or *SETD1B* mRNA expression were snap-frozen in liquid nitrogen within 30 min after surgical resection. This study was approved by Ethics Committee of the First Affiliated Hospital of Sun Yat-sen University.

### Processing of gene expression omnibus (GEO) and the gene expression profiling interactive analysis (GEPIA2) data

The gene expression profiles of GSE107943 were downloaded from the GEO database. The database consisted of 27 pairs of ICC cancer and adjacent non-cancerous tissues samples. The mRNA expression of *METTL3* were compared between the two groups.

The GEPIA2 database consisted of 36 ICC cancer and 9 adjacent non-cancerous tissues samples. We compared the mRNA expression of *METTL3* and *SETD1B* between the two groups. The correlation of *METTL3* and *SETD1B* expression in 36 ICC tissues were also analyzed.

### Cell culture and transfection

Two human ICC cell lines HuCC-T1 and HCCC-9810 were obtained from Cellcook Co., Ltd. (Guangzhou, China). The ICC cell lines HuCC-T1 and HCCC-9810 were cultured in RPMI 1640 medium (Gibco, USA) supplemented with 10% of fetal bovine serum (FBS, Gibco) in an incubator at 37 °C with 5% CO_2_.

The transfection of siRNA and shRNA were performed as described previously [30].

### Reagents

Targeting human *IFIT2* and non-targeting control small interfering RNAs (siRNAs) were purchased from RiboBio Co., Ltd. (Guangzhou, China). The *METTL3*, *YTHDF2* and *SETD1B* short hairpin RNA (shRNA) sequence were purchased from Sangon Biotech Co., Ltd (Shanghai, China), and then ligated into pLVX-Puro-GFP empty vector to construct shRNA plasmid. The CPI-455 (#S8287) was purchased from Selleck Chemicals (Houston, TX, USA). The STM2457 (#T9060) was purchased from TOPSCIENCE Co. Ltd (Shanghai, China). All sequences for RNA interference and primers sequences used for the experiments were listed in Supplementary Table 4.

### Western blotting

Western blotting was performed as described previously [30]. The anti-METTL3 antibody (#ab195352) and anti-Ki67 (#ab156956) were purchased from Abcam. The anti-IFIT2 antibody (#DF8962) was purchased from Affbiotech. The anti-H3K4me3 antibody (#39160) was purchased from Active Motif. The anti-β-actin antibody (#4970) and anti-rabbit IgG, HRP-linked antibody (#7071) was purchased from Cell Signaling Technology. The anti-m^6^A antibody (#202003) was purchased from Synaptic Systems. The anti-histone H3 antibody (#ab21054) was purchased from Abcam. The anti-YTHDF2 antibody (#24744-1-AP) was purchased from Proteintech.

### Dual luciferase reporter assay

The 3’UTR regions of *IFIT2* were amplified by PCR from cDNA and cloned into the psicheck-2 vector to construct WT-IFIT2 dual-luciferase reporter plasmid. The 3’UTR fragment which mutated at *IFIT2* m^6^A site (RRACH → RRGCH) was synthesized by Sangon Biotech Co., Ltd and cloned into the psicheck-2 vector to construct Mut-*IFIT2* dual-luciferase reporter plasmid.

For dual luciferase reporter assay, the ICC cells with different treatment were transfected in 6-well plates with dual-luciferase reporter plasmid. At 48 h, the luciferase activity was measured by the Dual-luciferase Reporter Assay System (Promega) according to manufacturer’s instructions.

### RNA decay assays

ICC cells were seeded in 6-well plate and treated with Actinomycin D (5 μg/ml, #A9415, Sigma–Aldrich, USA) for indicated time. The total RNA was extracted with TRIzol reagent (Life Technologies, USA) and processed RT-qPCR analysis.

### Animal experiments

4-week-old female BALB/c nude mice were used for HuCC-T1 tumor xenograft models and 4-week-old female B-NDG^®^ mice (Biocytogen, Beijing, China) were used for HCCC-9810 tumor xenograft models. 1 × 10^7^ HuCC-T1 or HCCC-9810 cells were resuspended in 100 ul PBS with Matrigel (1:1), and injected into the right flank of mice (*n* = 6/group). The tumor volume was measured every 4 days by caliper. The formula for volume is length × width^2^/2. After 28 days of implantation, the mice were executed. The xenograft tumors were removed, photographed and weighed. The proliferation index was detected by IHC staining of KI67. The apoptosis cells in tumors were detected by terminal deoxynucleotidyl transferase dUTP nick end labeling (TUNEL) assay. All animals used in the experiment were approved by the First Affiliated Hospital of Sun Yat-sen University ([2020] No. 382).

### Immunohistochemical (IHC) staining

This staining assay was performed as described previously [30]. The IHC score was calculated as staining intensity (negative, 0; mild, 1; moderate, 2; severe, 3) multiplying staining area (negative, 0; ≤30%, 1; >30 and ≤60%, 2; >60%, 3) by two experienced pathologists independently. The median scores for METTL3 and IFIT2 were three and four, respectively. For IHC staining of METTL3, IHC score >3 was defined as high METTL3 (*n* = 56), and IHC score ≤3 was defined as low METTL3 expression (*n* = 40). For IHC staining of IFIT2, IHC score >3 was defined as high IFIT2 (*n* = 34), and IHC score ≤3 was defined as low METTL3 expression (*n* = 62).

### ChIP-qPCR

ICC cells were crosslinked in 1% formaldehyde (final concentration) for 10 min at room temperature and followed by 250 mM Glycine quenching. Cell lysates were processed using the SimpleChIP Enzymatic Chromatin IP Kit (Cell Signaling Technology) according to manufacturer’s instructions. The H3K4me3 antibody was used for immunoprecipitation at a dilution factor of 1:1000. ChIP DNA was purified, and subsequent qPCR was conducted using the 2 × ChamQ Universal SYBR qPCR Master Mix (Vazyme). A flank region with no signal was used as a negative control.

### RNA-seq

HuCC-T1 cells, infected with lentiviruses expressing sh*METTL3*-1 and sh*METTL3*-2, were harvested at 48 h post-infection, followed by RNA extraction using TRIzol solution (Life Technologies). The cDNA library was prepared by Novogene (Beijing, China). The paired-end reads were generated by the Illumina^®^ HiSeq 2500 platform supplied by Novogene. An R package, DESeq, was used to quantify transcription levels and identify differentially expressed genes, using a cut-off of *P* < 0.05.

### MeRIP-seq and MeRIP-qPCR

Total RNA was isolated from indicated HuCC-T1 and HCCC-9810 cells, and the mRNA was further purified using Dynabeads mRNA Purification Kit (61006, Invitrogen, USA). After fragmentation with RNA fragmentation reagent (AM8740, Invitrogen, USA), the anti-m^6^A antibody was used for immunoprecipitated. Both input and immunoprecipitation RNA samples were then subjected to the sequencing libraries preparation using NEBNext Ultra RNA Library Prep Kit for Illumina and submitted for sequencing on Illumina HiSeq 2500 or MeRIP-qPCR analysis.

### CLIP-seq

In short, we induced the covalent cross-link between the protein and the directly bound RNA by ultraviolet irradiation. In this way, RNA-protein crosslinking is achieved. After cross-linking, the RNA is partially hydrolyzed to reduce the bound RNA fragments to a “footprint” size (usually about 30–50 nt), which can be cloned by RNA adaptor ligation and reverse transcription (RT)-PCR amplification. Then sequence these PCR products on a high-throughput platform, which was completed on Illumina Hi-Seq. The RNA-IP performed as described previously [31].

### RIP-seq or RIP-qPCR

HuCC-T1 and HCCC-9810 Cells were seeded in a 150-mm dish at a density of 1 × 10^6^ cells/ml. The cells were harvested in lysis buffer. The anti-YTHDF2 antibody was used for immunoprecipitated in a dilution of 1:100. The RNA of input and immunoprecipitated samples were isolated with the TRIzol reagent and subjected to sequencing using Illumina HiSeq 2500 platform supplied by Novogene or qPCR analysis. For qPCR analysis, a flank region with no signal was used as a negative control.

### Statistical analysis

The statistical analysis of the data was carried out in SPSS 22.0 software and GraphPad Prism 9 software. The data were expressed as mean ± standard deviation and compared by *t* test, Wilcoxon test, or Chi square test. DFS and OS were measured by Kaplan–Meier method, and the differences between groups were evaluated by log rank test. The independent predictive factors were determined by the cox proportional hazard model. All the statistical analyses, *p* < 0.05 were considered statistically significant.

## Supplementary information

Supplemental
