# Supplementary material for: METTL3 promotes intrahepatic cholangiocarcinoma progression by regulating IFIT2 expression in an m6A-YTHDF2-dependent manner
Source: Oncogene. 2022 Jan 29;41(11):1622–33. doi: 10.1038/s41388-022-02185-1 (PMC8913368; doi:10.1038/s41388-022-02185-1)
Supplement: Supplementary file 1 — Supplemental [file 41388_2022_2185_MOESM1_ESM.doc]

**Supplemental information**

**METTL3 promotes intrahepatic cholangiocarcinoma progression by regulating IFIT2 expression in an m^6^A-YTHDF2-dependent** **manner**

Qiong-Cong Xu^1#^, Yi-Chih Tien ^1#^, Yin-Hao Shi^1#^, Siyun Chen^3^, Ying-Qin Zhu^1^, Xi-Tai Huang^1^, Chen-Song Huang^1^，Wei Zhao^2,3^* and Xiao-Yu Yin^1^*

**Contents**

**Supplemental Figure 1-5**

**Supplemental Table 1-4**

**Supplemental Methods**


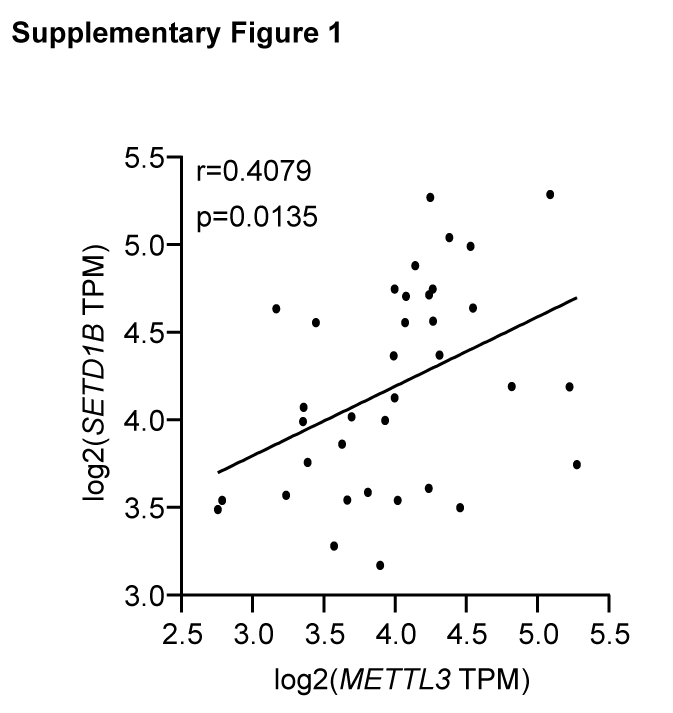


**Figure S1.** **Correlation analysis of METTL3 expression with SETD1B expression in GEPIA2 database.**

**
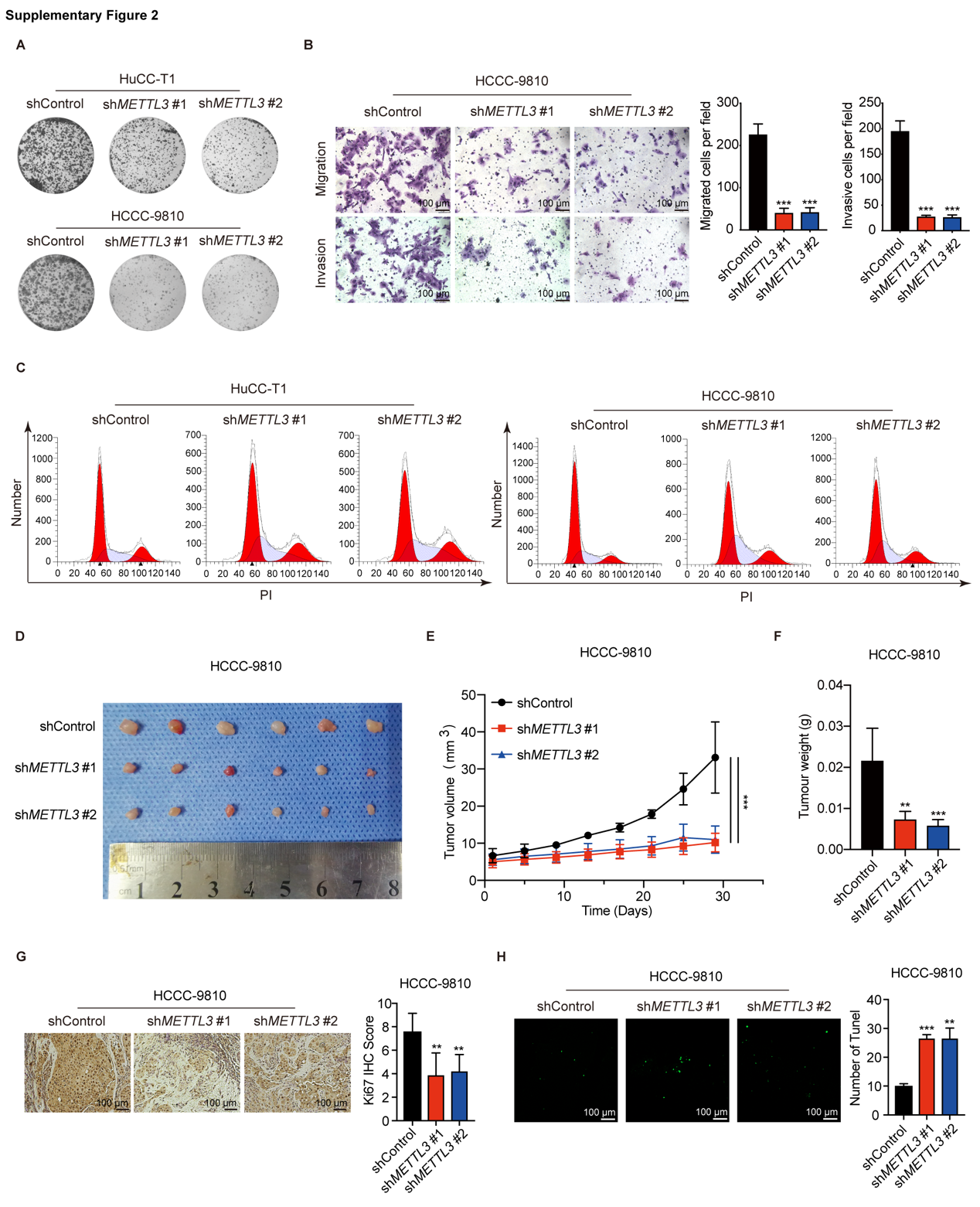
**

**Figure S2. METTL3 knockdown inhibits ICC progression.**

A. Representative images of colony forming assays after sh*METTL3*-transfection in HuCC-T1 and HCCC-9810 cells.

B. Cell migration ability and cell invasion ability after sh*METTL3*-transfection in HCCC-9810 cell.

C. Cell cycle analysis of HuCC-T1 and HCCC-9810 cells transfected with shControl or sh*METTL3*.

D. Xenograft tumors of HCCC-9810 in each group were shown. The mice were sacrificed 28 days post-injection.

E. Tumor growth curves in HCCC-9810 xenograft tumors with different treatments. Tumor volume was calculated every 4 days.

F. Tumor weight of knockdown or control groups in HCCC-9810 xenograft tumors was measured.

G. Representative IHC staining of Ki67 in HCCC-9810 xenograft tumors with different treatments.

H. Representative images of TUNEL analysis in HCCC-9810 xenograft tumors with different treatments.

The results are presented as mean ± SD of three independent experiments. **P*<0.05, ***P*<0.01, ****P*<0.001, according to a Student’s t-test.

**
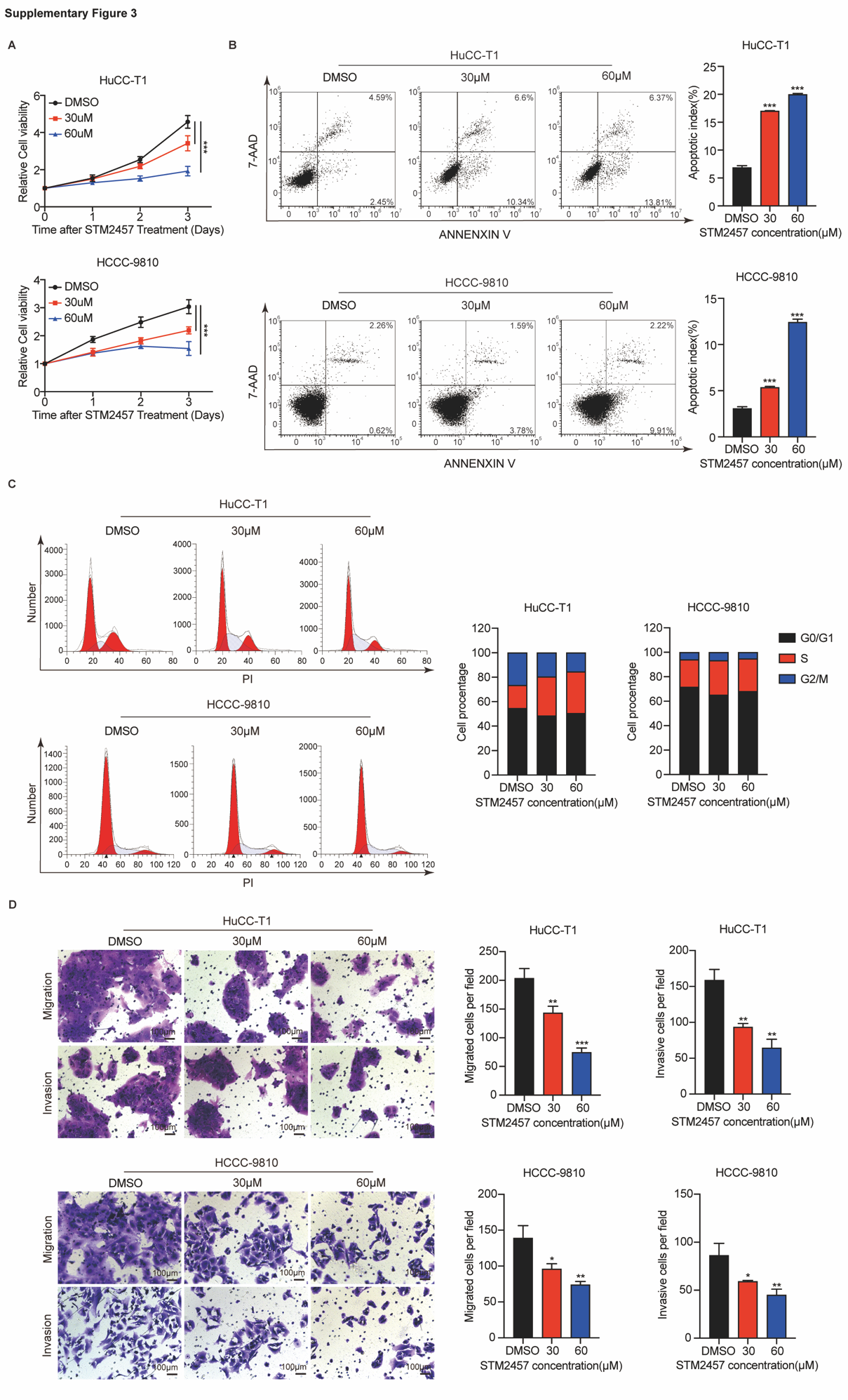
**

**Figure S3. The effects of METTL3 inhibitor STM2457 on ICC progression.**

A. Cell growth curve of HuCC-T1 and HCCC-9810 cells treated with different concentrations of STM2457 or DMSO.

B. Apoptosis analysis of HuCC-T1 and HCCC-9810 cells treated with different concentrations of STM2457 or DMSO.

C. Cell cycle analysis of HuCC-T1 and HCCC-9810 cells treated with different concentrations of STM2457 or DMSO.

D. Cell migration ability and cell invasion ability of HuCC-T1 and HCCC-9810 cells treated with different concentrations of STM2457 or DMSO.

**
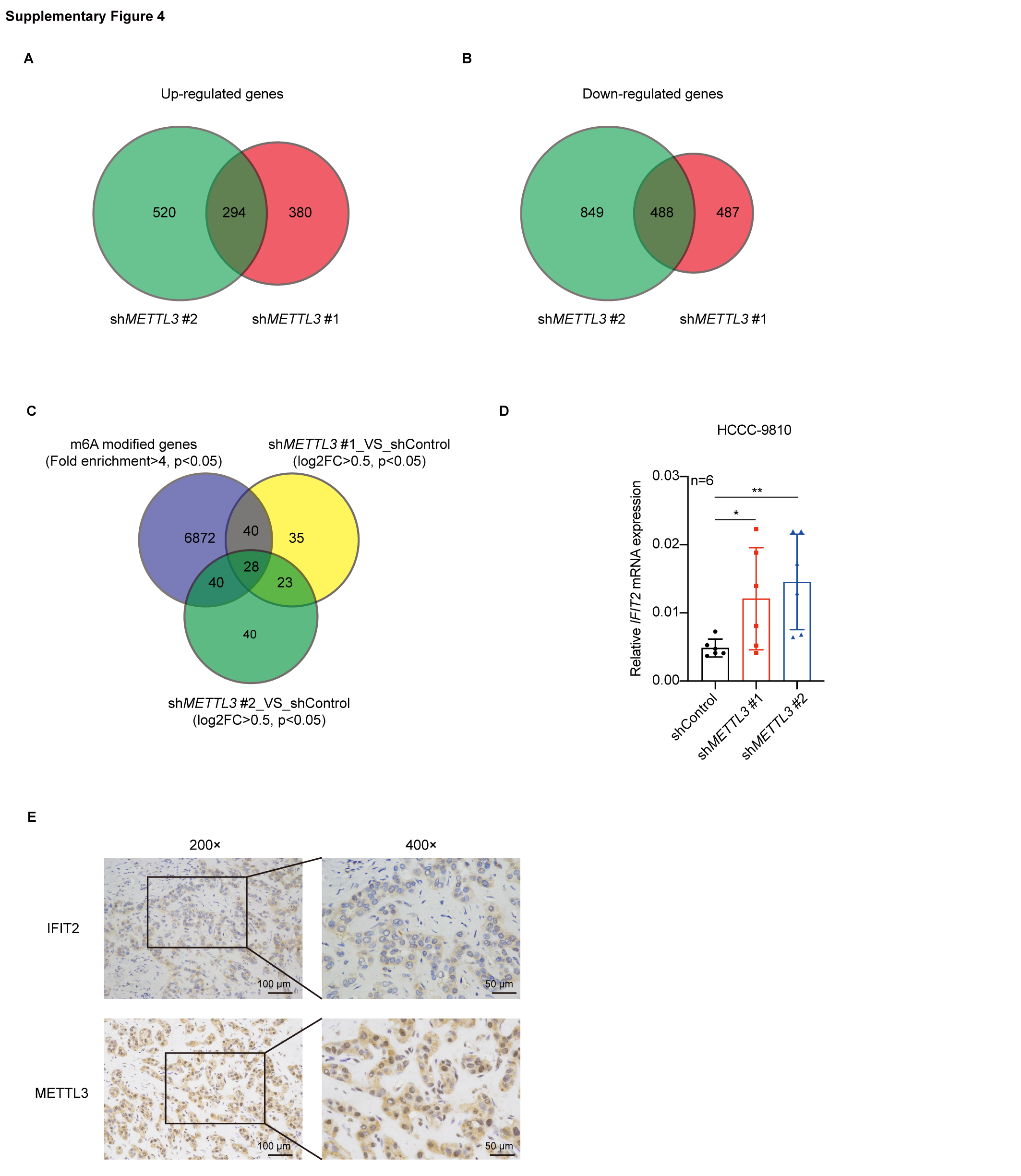
**

**Figure S4. IFIT2 is the downstream target of METTL3.**

A. Venn diagrams show the significantly upregulated genes after sh*METTL3*-transfection in HuCC-T1 cell.

B. Venn diagrams show the significantly downregulated genes after sh*METTL3*-transfection in HuCC-T1 cell.

C. Venn diagram shows the genes with m^6^A modification (Fold enrichment >4 and *P* <0.05) in both HuCC-T1 and HCCC-9810 cells and differentially expressed (Log2FC > 0.5 and *P* <0.05) in HuCC-T1 cells after METTL3 silencing.

D. RT-qPCR analysis of *IFIT2* mRNA expression in HCCC-9810 xenograft models after METTL3 knockdown or not.

E. Representative IHC staining images of ICC tumors expressing IFIT2 and METTL3.

**
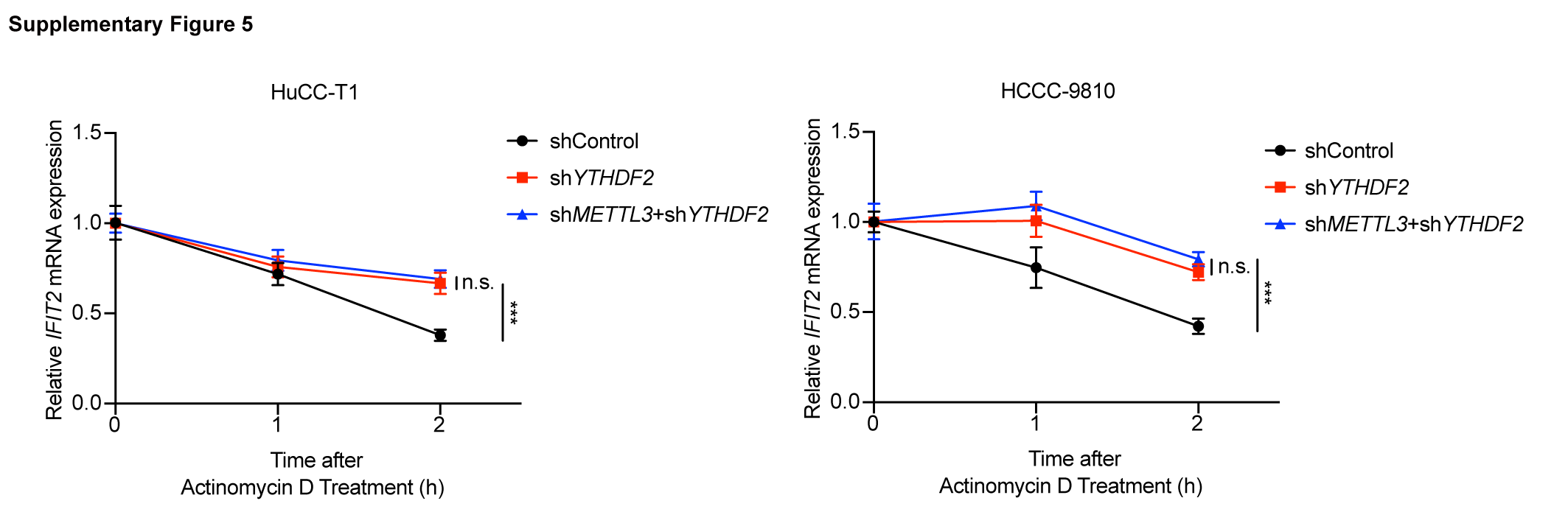
**

**Figure S5. RT-qPCR analysis of IFIT2 after actinomycin D treatment in YTHDF2-KD, YTHDF2-KD +METTL3-KD or Control HuCC-T1 and HCCC-9810 cells.**

**Supplementary Table 1. Correlation between METTL3 expression and clinicopathological characteristics in 96 ICC patients.**

| Characteristics | | Number of patients | | | *P*-value^*^ |
| --- | --- | --- | --- | --- | --- |
|  |  | Low METTL3 expression | High METTL3 expression | |  |
| Gender | Male | 26 | 27 | 0.145 | |
|  | Female | 14 | 29 |  |  |
| Age | ≤60 | 24 | 29 | 0.533 | |
|  | ＞60 | 16 | 27 |  |  |
| Tumor size | ≤5cm | 21 | 16 | 0.021 | |
|  | ＞5cm | 19 | 40 |  |  |
| CA19-9, U/ml | ≤37 | 17 | 28 | 0.219 | |
|  | ＞37 | 23 | 28 |  |  |
| TBIL, µmol/L | ≤ 34.4 | 30 | 48 | 0.198 | |
|  | >34.4 | 10 | 8 |  |  |
| TNM stage | I/II | 23 | 20 | 0.040 | |
|  | III/IV | 17 | 36 |  |  |
| Lymphatic metastasis | Positive | 9 | 22 | 0.121 | |
|  | Negative | 31 | 34 |  |  |
| Distant metastasis | Positive | 5 | 11 | 0.415 | |
|  | Negative | 35 | 45 |  |  |
| Vascular invasion | Positive | 6 | 11 | 0.600 | |
|  | Negative | 34 | 45 |  |  |
| Nerve invasion | Positive | 5 | 8 | 1.000 | |
|  | Negative | 35 | 48 |  |  |

^*^ Chi-square test.

**Supplementary Table 2. Univariate and multivariate cox analyses to evaluate the predictors for OS of ICC.**

| Variables | OS | | | |
| --- | --- | --- | --- | --- |
|  | Univariate analysis | | Multivariate analysis | |
|  | HR (95%CI) | *p* value | HR (95%CI) | *p* value |
| IHC score (≤3 vs.＞3) | 2.259 (1.334-3.825) | 0.002 | 2.105 (1.246-3.557) | 0.005 |
| Tumor size (cm) (≤5 vs. ＞5) | 2.222 (1.302-3.792) | 0.003 | 2.366 (1.319-4.245) | 0.004 |
| Vascular invasion (no vs. yes) | 4.140 (2.269-7.556) | <0.001 | 2.903 (1.580-5.335) | 0.001 |
| Nerve invasion (no vs. yes) | 2.283 (1.203-4.333) | 0.012 | 3.140 (1.569-6.285) | 0.001 |
| Gender (male vs. female) | 0.737 (0.448-1.213) | 0.230 |  |  |
| Age (y) (<60 vs. ≥60) | 1.097 (0.668-1.800) | 0.715 |  |  |
| CA19-9 (U/ml) (<35 vs. ≥35) | 1.989 (1.193-3.316) | 0.008 |  |  |
| TNM stage (I/II vs. III/IV) | 2.658 (1.579-4.474) | <0.001 |  |  |
| Lymphatic metastasis (no vs. yes) | 2.629 (1.549-4.461) | <0.001 |  |  |
| Distant metastasis (no vs. yes) | 2.054 (1.084-3.891) | 0.027 |  |  |
| TBIL (µmol/L) (≤ 34.4 vs. >34.4) | 0.948 (0.515-1.746) | 0.865 |  |  |

**Supplementary Table 3. The clinicopathological characteristics of 96 ICC patients used in our study.**

| Characteristics | Values |
| --- | --- |
| Age, year (mean ± SD) | 57.31 ± 11.6 (21-79) |
| Gender (male/female) | 53/43 |
| Tumor size, cm (≤5/＞5) | 37/59 |
| CA19-9, kU/L (≤37/＞37) | 45/51 |
| TBIL, µmol/L (≤ 34.4/＞34.4) | 78/18 |
| TNM stage (I&II/III&IV) | 66/30 |
| Lymphatic metastasis (Negative, Positive) | 65/31 |
| Distant metastasis (Negative, Positive) | 16/80 |
| Vascular invasion (Negative, Positive) | 79/17 |
| Nerve invasion (Negative, Positive) | 83/13 |
| Tumor recurrence, months (mean ± SD) | 17.1±22.8 (1-118) |
| Overall survival, months (mean ± SD) | 21.7 ± 22.8 (1-118) |

**Supplementary Table 4. Sequences of shRNA, siRNA and primers used for experiments in this study.**

| Names | Sequences |
| --- | --- |
| METTL3 shRNA-1 | GCTGCACTTCAGACGAATTAT |
| METTL3 shRNA-2 | GCCAAGGAACAATCCATTGTT |
| SETD1B shRNA-1 | GGAGATTACCTATGACTATAA |
| SETD1B shRNA-2 | GCCGCCACGAACATCATTATG |
| YTHDF2 shRNA-1 | ccacaggcaaggcccaataat |
| YTHDF2 shRNA-2 | TCTGGATATAGTAGCAATTAT |
| IFIT2 siRNA-1 | GACTGGCAATAGCAAGCTA |
| IFIT2 siRNA-2 | GACTGAGTTTCAGAATCGT |
| GAPDH-primer-F | GGAGCGAGATCCCTCCAAAAT |
| GAPDH-primer-R | GGCTGTTGTCATACTTCTCATGG |
| METTL3-primer-F | TTGTCTCCAACCTTCCGTAGT |
| METTL3-primer-R | CCAGATCAGAGAGGTGGTGTAG |
| YTHDF2-primer-F | AGCCCCACTTCCTACCAGATG |
| YTHDF2-primer-R | TGAGAACTGTTATTTCCCCATGC |
| IFIT2-primer-F | AAGCACCTCAAAGGGCAAAAC |
| IFIT2-primer-R | TCGGCCCATGTGATAGTAGAC |
| SETD1B-primer-F | CTGGGTCTACCATCCCTCCA |
| SETD1B-primer-R | CTTCCGGAACTTGAGCTGGT |
| primer-F for H3K4me3 ChIP-qPCR | CTTTGGCGCTTTGTTCCCTC |
| primer-R for H3K4me3 ChIP-qPCR  primer-F for NT H3K4me3 ChIP-qPCR  primer-R for NT H3K4me3 ChIP-qPCR | CACTAAATTGGGCACTGCGG  GATTCCAGTGCAGACCGACT  GAGATTCATGGCCCGAGTCC |
| primer-F for MeRIP-qPCR | GTTTGGAGTCTGGAAGCCTCA |
| primer-F for MeRIP-qPCR  primer-F for NT MeRIP-qPCR  primer-R for NT MeRIP-qPCR | TTCAGCAGTAGCCTAGTGGG  AGCGAAGGTGTGCTTTGAGA  GAGGGTCAATGGCGTTCTGA |

**Supplemental Methods**

**RT-qPCR**

The total RNA of indicated cells with different treatment was extracted using the TRIzol reagent (Invitrogen, USA). RNA reverse transcription was conducted with HiScript II Q RT SuperMix for qPCR (R223-01, Vazyme, Nanjing, China) according to the manufacturer’s instructions. The ChamQ Universal SYBR qPCR Master Mix（Q711-02, Vazyme）was used for qPCR analysis. The relative expression levels were detected by QuantStudio 6 Flex Real-Time PCR Systems (Applied Biosystems, USA) and analyzed according to 2^−ΔΔCT^.

**Cell viability assay and plate clone formation assay**

For the cell viability assay, 2 000 ICC cells with different treatment were seeded in the 96-well plate. The intensity of Luminescence was measured at 24, 48, and 72h using the CellTiter-Glo® Luminescent Cell Viability Assay (Promega, Madison, WI, USA) according to the manufacturer's instructions.

For the plate clone formation assay, 1 000 ICC cells with different treatment were seeded in the 6-well plate. After culturing for 14 days, the cells were fixed with 4% formaldehyde and then stained with crystal violet. The number of cell clones were then counted and analyzed.

**Invasion and Migration Assays**

For invasion and migration assays, 40 000 cells with different treatment were resuspended in 300 µL medium without FBS and seeded in the transwell chamber (Falcon, USA) with (for invasion assay) or without (for migration assay) Matrigel (Corning, USA). The transwell chambers were then placed in 24 well plates containing 800 µL medium with 10% of FBS. At 24h, the cells in the lower surface of the transwell chamber were fixed with 4% formaldehyde and then stained with crystal violet. The number of cells were then counted and analyzed.

**Apoptosis assay**

After 48h of different treatment with ICC Cells, the cells were collected and analyzed using Annexin V-FITC / PI Apoptosis Kit (#BB4101, BestBio, Shanghai, China) according to the manufacturer's instructions. After staining, the apoptosis rate was measured by flow cytometer (BD Bioscience)

**Cell cycle assay**

The ICC cells were processed by different lentivirus. Collect the cells 48 hours after transfection and use the cell cycle staining kit (#BB4104, BestBio) to carry out the cell cycle process. The data was detected by flow cytometry and analyzed by ModFit LT 4.1 software (Verity Software House).
